# Synergistic effects of plasma S100B and MRI measures of cerebrovascular disease on cognition in older adults

**DOI:** 10.1007/s11357-024-01498-1

**Published:** 2025-02-05

**Authors:** Colleen Pappas, Christopher E. Bauer, Valentinos Zachariou, T. J. Libecap, Beatriz Rodolpho, Tiffany L. Sudduth, Peter T. Nelson, Gregory A. Jicha, Anika MS Hartz, Xingfeng Shao, Danny J. J. Wang, Brian T. Gold

**Affiliations:** 1 Department of Neuroscience, College of Medicine, University of Kentucky, Lexington, KY 40536 USA; 2 Department of Behavioral Science, College of Medicine, University of Kentucky, Lexington, KY 40536 USA; 3 Sanders Brown Center On Aging, University of Kentucky, Lexington, KY 40536 USA; 4 Department of Pathology and Laboratory Medicine, University of Kentucky, Lexington, KY 40536 USA; 5 Department of Neurology, University of Kentucky, Lexington, KY 40536 USA; 6 Department of Pharmacology & Nutritional Sciences, University of Kentucky, Lexington, KY 40536 USA; 7 Keck School of Medicine, University of Southern California, Los Angeles, CA 90033 USA; 8 Department of Radiology, College of Medicine, University of Kentucky, Lexington, KY 40536 USA; 9 Magnetic Resonance Imaging and Spectroscopy Center, University of Kentucky, Lexington, KY 40536 USA

**Keywords:** Vascular contributions to cognitive impairment and dementia, Blood–brain barrier, S100B, Diffusion, White matter hyperintensities, Memory

## Abstract

There is growing interest in studying vascular contributions to cognitive impairment and dementia (VCID) and developing biomarkers to identify at-risk individuals. A combination of biofluid and neuroimaging markers may better profile early stage VCID than individual measures. Here, we tested this possibility focusing on plasma levels of S100 calcium-binding protein B (S100B), which has been linked with blood–brain-barrier (BBB) integrity, and neuroimaging measures assessing BBB function (water exchange rate across the BBB (k_w_)) and cerebral small vessel disease (white matter hyperintensities (WMHs)). A total of 74 older adults without dementia had plasma samples collected and underwent cognitive assessment. A subsample had neuroimaging data including diffusion prepared pseudo-continuous arterial spin labeling (DP-pCASL) for assessment of BBB k_w_ and T2-weighted fluid-attenuated inversion recovery (FLAIR) for quantification of WMHs. Results indicated that higher plasma S100B levels were associated with poorer episodic memory performance (*β* = − .031, SE = .008, *p* < .001). Moreover, significant interactions were observed between plasma S100B levels and parietal lobe BBB k_w_ (interaction *β* = .095, SE = .042, *p* = .028) and between plasma S100B levels and deep WMH volume (interaction *β* = − .025, SE = .009, *p* = .007) for episodic memory. Individuals with the poorest memory performance showed both high plasma S100B and either low BBB k_w_ in the parietal lobe or increased deep WMH burden. Taken together, our results provide support for the combined use of biofluid and neuroimaging markers in the study of VCID.

**Supplementary Information:**

The online version contains supplementary material available at 10.1007/s11357-024-01498-1.

## Introduction

Vascular contributions to cognitive impairment and dementia (VCID) are very common in older adults and being increasingly studied [1, 2]. The recognition that VCID is a significant contributor to age-related cognitive declines has led to calls for adding a vascular component to the canonical amyloid/tau/neurodegeneration (ATN) framework [3, 4]. VCID results from cerebrovascular disease (CVD) and can be caused by insults to endothelial cells, pericytes, and astrocytes [1, 5]. As these cells comprise the blood–brain barrier (BBB), a major focus is emerging toward assessing BBB integrity in relation to VCID [6, 7].

As with other neurodegenerative processes, VCID is multifactorial and will likely require multiple biomarkers for early identification and stratification. It is therefore important to study combinations of potential markers, particularly across multiple modalities such as biofluid and neuroimaging, that may better capture components of VCID than any single marker in isolation [8, 9]. Blood-based biomarkers are relatively accessible and inexpensive, making them potentially attractive markers [10, 11], particularly if they can be linked with cognitive function and index mechanisms not gauged by neuroimaging measures.

One biofluid marker that may help in identifying early-stages of VCID is blood-based S100 calcium-binding protein B (S100B). In the central nervous system (CNS), S100B is primarily expressed by glial cells, including astrocytes, whose endfeet form a portion of the BBB [12]. Serum S100B has often been used as a marker for traumatic brain injury, and in turn, BBB leakage [13–16]. Work with mouse models further substantiates the involvement of S100B in BBB integrity, as S100B knockout mice show increased BBB permeability [17]. Elevated blood-based S100B levels have also been observed among individuals with AD [18] and cerebral small vessel disease (cSVD) [19].

Considering neuroimaging measures of CVD, a recently developed and validated method called diffusion prepared, pseudo-continuous arterial spin labeling (DP-pCASL) is thought to index BBB function [20–22]. DP-pCASL estimates cerebral perfusion in multiple brain compartments by introducing a small diffusion gradient that separates the pCASL signal into labeled water in the capillaries versus brain tissue based on their distinctive diffusion coefficients (high in capillaries and low in tissue) [23]. A single-pass approximation model [24] is then used to assess the water exchange rate (k_w_) between the capillaries and parenchyma [20, 21]. BBB k_w_ has been associated with cognitive performance [25, 26], although our recent work suggests this relationship may be region-specific [27].

While DP-pCASL represents a promising new method to assess BBB function, white matter hyperintensity (WMH) volume assessed via T2-weighted fluid-attenuated inversion recovery (FLAIR) imaging remains a widely used and clinically relevant neuroimaging metric of CVD [28, 29]. WMHs reflect axonal degeneration, demyelination, and gliosis [30, 31] and can be indicative of BBB dysfunction [32]. Assessing both novel and classic neuroimaging biomarkers of CVD in concert with S100B may provide further insight into VCID-related cognitive function.

Toward that goal, this study aimed to test the relationship between plasma S100B levels and both memory and executive function in older adults without dementia. We also examined associations between plasma S100B levels and neuroimaging measures of CVD, using a novel marker of BBB function (water exchange rate [k_w_]) and a classic marker of CVD (WMHs). Finally, and most importantly, moderation analyses were conducted to identify the potentially synergistic effects of S100B and neuroimaging measures of CVD on cognitive function in older adults.

## Materials and method

### Participants

Participants in the current study were recruited from the Sanders-Brown Center on Aging (SBCoA) at the University of Kentucky [33] and were enrolled in the University of Kentucky Alzheimer’s Disease Research Center (UK-ADRC) Longitudinal Cohort Study. The UK-ADRC longitudinal cohort inclusion criteria were a minimum of 60 years of age, cognitive and neurological normality at the enrollment examination (based on clinical consensus diagnosis performed by the UK-ADRC Clinical Core), designated informant for structured interviews, willingness to undergo annual cognitive testing, blood draw, and physical and neurological examinations. While cognitive normality is an inclusion requirement, participants who develop cognitive impairment after enrollment continue to be followed in the UK-ADRC Longitudinal Cohort. Exclusion criteria for the UK-ADRC longitudinal cohort were a history of head injury, major psychiatric illness or current substance abuse, medical illnesses that are nonstable, impairing, or that affect the CNS, chronic infectious diseases, stroke or transient ischemic attack, encephalitis, meningitis, or epilepsy.

Additional exclusion criteria for the present study were MRI-related contraindications (i.e., pacemakers, metal fragments, metal implants, claustrophobia) or brain abnormalities discovered upon imaging. Additional inclusion criteria for the present study were having a blood draw, MRI scan, and neuropsychological exam collected within 1 year of each other. In the present study, we excluded for dementia but not for mild cognitive impairment. The study was approved by the University of Kentucky Institutional Review Board, and all participants provided written informed consent.

### Plasma sample collection and S100B quantification

A fasting blood draw was performed via venipuncture and stored in ethylenediaminetetraacetic acid (EDTA) tubes at the Sanders Brown Center on Aging as outlined in prior studies [34, 35]. Plasma samples were processed, aliquoted, and stored in a − 80 °C freezer until ready for biomarker quantification. Plasma samples were transferred to the University of Kentucky Center for Clinical and Translational Science (CCTS) Biomarker Analysis Lab for further processing. Plasma levels of S100B were assessed via an electrochemiluminescence immunoassay Meso Scale Discovery R-Plex kit for human S100B (Meso Scale Design, Rockville, MD; catalog #K1512ER-2). Standard protocol was followed and samples were not diluted following optimization testing. All samples were run in duplicate. Samples were read with a MESO QuickPlex instrument, and S100B was measured in pg/mL. The lower detection limit was 0.276 pg/mL, and the upper detection limit was 5000 pg/mL for the assay.

### Neuropsychological testing

Neuropsychological testing was performed at the UK ADRC by trained neuropsychologists using the National Alzheimer’s Coordinating Center’s Uniform Data Set, version 3 [36]. Scores on the Montreal Cognitive Assessment [MoCA; 37] are reported as a measure of global cognitive function. For analytic purposes, we focused on two domains of cognition known to undergo significant age-related declines: episodic memory and executive function. Composite scores were calculated from several neuropsychological tests for episodic memory and executive function domains as described below.

Three tests were used to create the episodic memory composite, which included the Craft Story immediate and delayed recall (paraphrase score) and the Benson complex figure delayed recall (total score). Each test was individually z-scored to the sample mean and then averaged together [38]. The executive function composite consisted of the following tests: category fluency (animals, vegetables), phonemic fluency (letter F, letter L), digit span backward, and trail making test A and B. Correct responses were recorded for category fluency, phonemic fluency, and digit span backward, and the correct number of lines per minute was calculated for the trail-making tests. The executive function composite was then created using factor score analysis as described by Staffaroni and colleagues [39]. Higher scores indicate better performance for both episodic memory and executive function composites.

### MRI acquisition

MRI scans were performed using a 3 Tesla Siemens Prisma MRI scanner with a 64-channel head coil at the University of Kentucky’s Magnetic Resonance Imaging and Spectroscopy Center (MRISC). For the purposes of this study, three sequences were used: (1) a 3D, T1-weighted magnetization-prepared rapid gradient echo (T1) sequence; (2) a 3D gradient-and-spin-echo (GRASE) diffusion-prepared pseudo-continuous arterial spin labeling (DP-pCASL) sequence; and (3) a 3D fluid-attenuated inversion recovery (FLAIR) sequence. Additional sequences were collected during the scanning session but do not pertain to scientific questions posed in the current study and are not described here.

The 3D T1-weighted, magnetization-prepared rapid gradient echo (MPRAGE) sequence covering the whole brain was acquired in the sagittal plane using a generalized autocalibrating partial parallel acquisition (GRAPPA) acceleration factor = 2, with 1 mm^3^ spatial resolution. Other T1-weighted parameters differed slightly based on whether participants were enrolled in an investigator-initiated MRI study (NIH/NIA R01 AG055449) [Multiecho MPRAGE (ME-MPRAGE); 256 × 256 × 176 mm acquisition matrix (176 slices), repetition time (TR) = 2530 ms, four echoes, first echo time (TE1) = 1.69 ms, echo time spacing (ΔTE = 1.86 ms), flip angle = 7°, scan duration = 5.88 min (*n* = 32)] or the UK-ADRC Longitudinal Cohort Study, which uses a Standardized Centralized Alzheimer’s Related Neuroimaging (SCAN) compatible T1-weighted sequence [MPRAGE: 256 × 240 × 208 mm acquisition matrix (208 slices), TR = 2300 ms, TE = 2.98 ms, flip angle = 9°, scan duration = 5.20 min (*n* = 42)].

The GRASE DP-pCASL scan sequence was collected using the following parameters: field of view = 224 mm, matrix size = 64 × 64, 12 slices (10% oversampling), resolution = 3.5 × 3.5 × 8 mm, TR = 4 s, TE = 36.5 ms, label/control duration = 1500 ms, centric ordering, optimized timing of background suppression for gray matter (GM) and white matter (WM), scan duration = 10 min [40]. A multi-stage approach was used to measure arterial transit time (ATT) and k_w_. First, ATT was acquired with 15 repetitions during the flow encoding arterial spin tagging (FEAST) scan at post‐labeling delay (PLD) = 900 ms and diffusion weighting (*b*‐value) of 0 and 14 s/mm^2^ [23]. The k_w_ metric was calculated from scans acquired at PLD = 1800 ms and diffusion weighting *b* = 0 and 50 s/mm^2^ to measure the labeled blood arriving at the microvascular compartment. For each *b*‐value of the k_w_ scan, 20 repetitions were acquired. Cerebral blood flow (CBF) was measured at the 1800 ms PLD as described by Shao et al. [20].

A 3D FLAIR sequence was collected covering the whole brain using a generalized autocalibrating partial parallel acquisition acceleration factor in the sagittal plane. Other FLAIR parameters differed slightly based on participant enrollment in the investigator-initiated study (NIH/NIA R01 AG055449) [256 × 256 × 176 mm acquisition matrix (176 slices), resolution 1 mm^3^, TR = 5000 ms, TE = 338 ms, TI = 1800 ms, scan duration = 6.53 min (*n* = 32)] or were part of the UK-ADRC Longitudinal Cohort Study, which uses a SCAN compatible FLAIR sequence [256 × 256 × 160 mm acquisition matrix (160 slices), resolution 1 × 1 × 1.2 mm, TR = 4800 ms, echo time TE = 441 ms, TI = 1650 ms, scan duration = 5.55 min (*n* = 39)].

### MRI processing

#### T1-weighted image

FreeSurfer (version 6.0) was used to process the T1-weighted MPRAGE and ME-MPRAGE (averaged into a root mean square image) images with the recon–all option [41, 42]. The whole brain was skull stripped and then used for registration with the DP-pCASL and 3D FLAIR sequence. Estimated total intracranial volume (eICV) for each participant was also derived from the T1-weighted image for use as a covariate in subsequent analyses.

#### Diffusion-prepared pseudo-continuous arterial spin labeling

Using the methodology outlined by Shao and colleagues [20], the DP-pCASL data were processed with the Water Exchange Quantification Toolbox (version 1). In brief, control/label images were subtracted to obtain perfusion images following motion correction with SPM12 [43]. Principal component analysis was used to minimize temporal fluctuations [44]. Whole brain maps were created for k_w_, ATT, and CBF. The tissue and capillary compartments of the ASL signal were separated by a small diffusion gradient of 50 s/mm^2^ for the sequence. The k_w_ map was calculated by a total‐generalized‐variation [TGV; 45 regularized SPA model [24] using the tissue (or capillary) fraction of the ASL signal at the PLD of 1800 ms, incorporating ATT, T1 of arterial blood and brain tissue as inputs for the algorithm [20]. From prior research with CBF data, arterial blood T1 was calculated as 1.66 s [46].

**Fig. 1:**
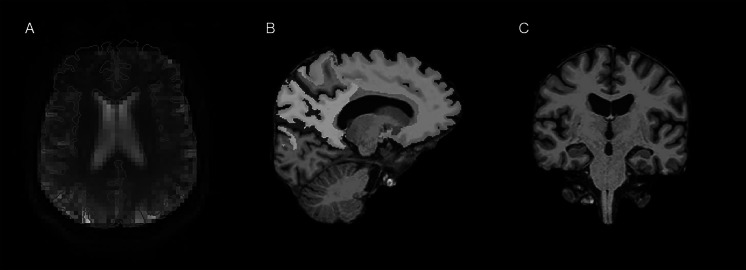
**Regions of interest for k**_w_** maps**. Regions of interest (ROIs) for k_w_ analyses displayed on a representative participant. **A** The diffusion prepared arterial spin labeling (DP-pcASL) M0 image with the T1-weighted image (red outline) aligned. **B** The frontal lobar (green), parietal lobar (yellow), and portions of precuneus (blue) masks on a mid-sagittal slice of the T1-weighted image. **C** The hippocampal (red) mask on a coronal slice of the T1-weighted image. The masks displayed in **B** and **C** were then resampled to the resolution of the M0 image

As detailed in our prior work, the T1-weighted image was then registered to the DP-pCASL M0 image [27]. A series of additional steps were needed for accurate registration because of the low resolution of the DP-pCASL images. First, the T1-weighted image was aligned with the center of the DP-pCASL M0 using the AFNI program @Align_Centers. Next, the center aligned T1-weighted image brain was registered to the DP-pCASL M0 by align_epi_anat.py using an edge-based approach to improve registration between the two images. The output matrix from the aforementioned process was then used to align the original images to one another. All T1-weighted images were visually inspected to ensure proper registration with the M0 image (see Fig. 1A for an example). In the case that registration was poor or failed, the nudge feature in FSLeyes was used and then subsequently applied to the alignment matrix. Once optimal registration was achieved, a binary mask of the whole brain T1-weighted image mask was created and then rescaled to the resolution of the DP-pCASL M0 image.

To determine k_w_ in specific regions of interest (ROIs), subcortical, cortical, and lobar masks were created in FreeSurfer from the recon-all option (see https://surfer.nmr.mgh.harvard.edu/fswiki/CorticalParcellation for documentation). The masks were registered to the DP-pCASL M0 image by applying the alignment matrix established for the whole brain in the T1-weighted image. To account for partial volume effects, all gray matter masks were eroded by one voxel. Masks were then rescaled to the resolution of the DP-pCASL M0 image. Five ROIs were selected due to regional associations with memory or executive function and based on prior work [26] which included the frontal lobe, parietal lobe, hippocampus, left precuneus, and right precuneus (see Fig. 1B and C). As the DP-pCASL sequence did not cover the entire brain, overlap between the T1-weighted image mask and the k_w_ map was estimated. A threshold of 90% coverage between the mask and k_w_ map was selected to ensure an unbiased measure of k_w_ across participants. ROIs for a given participant which fell below the selected threshold were not included in subsequent analyses.

Each k_w_ map was z-scored to the participant’s average whole brain k_w_ to standardize comparisons between subjects. A more conservative approach was selected to better determine region-specific effects of k_w_ that may be related to plasma S100B levels. The average z-scored k_w_ values for each ROI (frontal lobe, parietal lobe, hippocampus, left and right precuneus) were used for analyses.

#### Fluid-attenuated inversion recovery (FLAIR)—white matter hyperintensities

Whole brain WMH volumes were calculated as outlined by DeCarli and colleagues [47] via a validated 4-tissue segmentation method. Using FLIRT from FMRIB Software Library [FSL; 48], participants’ FLAIR images were registered to their skull stripped T1-weighted image (root mean square image). Next, any inhomogeneities in FLAIR images were corrected using a previously published local histogram normalization and aligned to a minimal deformation template [MDT; 47] using non-linear methods [49]. Bayesian probability techniques based on histogram fitting and prior probability maps were used to estimate WMHs in template space. WMHs were identified as voxels 3.5 SDs above the mean WM signal intensity from each participant’s estimated template space map. The WMHs in template space were then back transformed to participants’ native space FLAIR image for quantification.

**Fig. 2:**
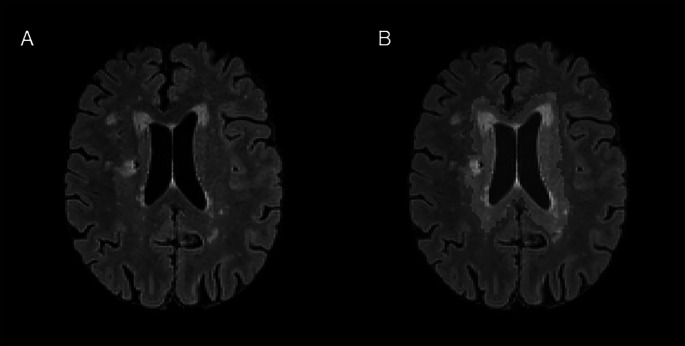
**White matter hyperintensity classification**. Periventricular and deep white matter hyperintensities (WMHs). A T2-weighted FLAIR image for a representative participant is displayed in **A**. The periventricular mask (yellow) and deep mask (red) are overlaid on the T2-image in **B**

WMHs were further categorized by location as periventricular or deep (see Fig. 2), which has been described in previous publications by our group [27, 50]. WMHs located within approximately 10 mm of the lateral ventricles were classified as periventricular, whereas WMHs outside this region were classified as deep [51]. Periventricular and deep ROIs were estimated using the validated Automatic Lateral Ventricle delIneatioN (ALVIN) mask, which is a group mask of the lateral ventricles derived from 275 healthy adults [52]. First, the participants’ skull-stripped T1 images were aligned to their respective FLAIR images using a local Pearson correlation cost function in AFNI. Additionally, a T1 whole brain mask was registered to the FLAIR image for future use with deep WMH calculation. The aligned T1 images were warped to MNI space [MNI ICBM152 1 mm, 6th generation atlas; 53] using a non-linear transformation, with the transformation matrix applied during this step created as output. As the ALVIN mask is in MNI space, the inverse transformation matrix was applied to move it into native space. Once the ALVIN mask was in native space for each participant, it was multiplied by the WMH mask to create the periventricular mask for a given participant. To create the deep WMH mask, the ALVIN mask was subtracted from the whole brain mask. The resulting mask was then multiplied by the WMH mask.

To ensure that erroneous voxels were not included in any WMH calculation, an automasking technique was used. First, each participant’s FLAIR image was transformed into MNI space using the transformation matrix created during T1 warping step. A union mask comprising WMHs in all locations across all participants in MNI space was then created by binarizing the average value in every voxel across all participant’s MNI-aligned WMH masks with AFNI’s 3dMean function. The union mask was clustered with the 3Dclust function to establish the minimum cluster threshold for WMH identification. The union mask was then binarized again, visually inspected, and manually edited where needed. Following editing, the mask was dilated by one voxel as to not exclude areas along the edge of the mask that may have otherwise been excluded due to partial voluming. The mask was transformed back into each participant’s native space and rescaled to the resolution of the FLAIR image. WMH volumes were extracted from the automasked FLAIR images and are reported as mm^3^. All WMH data was log transformed (WMH + 1 due to a zero-value present in data) to achieve normality.

### Statistical analyses

Multiple linear regression analyses were used to test the relationship between plasma S100B and the dependent variables of interest: cognition, BBB water exchange rate (k_w_), and WMHs. For models examining relationships between S100B and cognition, covariates included age, gender, and education. For the S100B-k_w_ associations, age, gender, and ROI size were used as covariates. Similarly, age, gender, and eICV were used as covariates for examining S100B-WMH relationships. To control for multiple comparisons, we applied a Holm-Bonferroni correction [54]. This method was employed separately for each dependent variable of interest grouping (i.e., cognition corrected for two tests, k_w_ corrected for five tests, WMHs corrected for three tests).

In addition to the multiple linear regression analyses, we also tested k_w_ and WMHs (separately) as possible moderators of the plasma S100B-cognition relationship. Analyses were completed with the PROCESS macro version 4.0 for SPSS, Model 1 [55]. Variables used for moderation were mean centered for ease of interpretation. S100B × kw interactions for episodic memory and executive function (dependent variables) were tested using age, gender, education, and ROI size as covariates. S100B × WMH interactions for episodic memory and executive function were tested using age, gender, education, and eICV as covariates. Significant interactions were further probed using the pick-a-point approach at low (16th percentile), mid (50th percentile), and high (84th percentile) values of the moderator variable. This is equivalent to moderator values at approximately the mean and one standard deviation above and below. As the moderation analyses are exploratory, we did not correct for multiple comparison.

Outliers greater than 3.29 standard deviations from the mean were excluded from the analyses. To test for multivariate outliers, Mahalanobis distance was also calculated [56]. The variance inflation factor (VIF) was used to assess multicollinearity, with values less than five considered acceptable [57]. All analyses were completed in SPSS version 28.0 (IBM Corp; Armonk, NY) and two-tailed alphas were set at *p* < 0.05.

## Results

### Participant characteristics

A total of 75 participants had plasma S100B and neuropsychological testing data collected within a 1-year time frame. One participant was an outlier for plasma S100B level. This resulted in a final sample size of 74 participants. Characteristics of the sample are outlined in Table 1. The participants were predominately female (58.1%) and had an average age of 74.7 years. Participants tended to be highly educated (Mean = 17 years) and had an average MoCA score of 26.4. The majority of the sample for this study was cognitively unimpaired (78.4% vs 21.6% with mild cognitive impairment) as determined by consensus diagnosis through the Clinical Core of the UK-ADRC.

**Table 1:** Participant characteristics (*n* = 74)

Characteristic	Mean	SD	No.(%)
Age (years)	74.67	1.58	
Gender (female)			43 (58.1)
Education (years)	16.99	2.69	
Cognitively unimpaired			58 (78.4)
MoCA Score	26.38	3.16	
S100B (pg/mL)	24.54	11.59	
Whole brain k_w_ (min^−1^)^a^	113.23	23.00	
WMH Total Volume (mm^3^)^b^	3.41	0.56	

Cognitive impairment was determined by a clinical consensus group. White matter hyperintensities are reported as log-transformed values

*MoCA* Montreal Cognitive Assessment, *SD* standard deviation, *WMH* white matter hyperintensities

^a^*n* = 62

^b^*n* = 71

A subsample of participants had neuroimaging measures. A total of 62 participants had DP-pCASL data collected that passed quality control and could be used for water exchange rate (k_w_) measurement. Seventy-two participants had FLAIR data collected that passed quality control, with one participant deemed an outlier and removed from analyses. This resulted in a sample size of 71 for the WMH-related analyses.

### Multiple linear regression: S100B and cognition

**Fig. 3:**
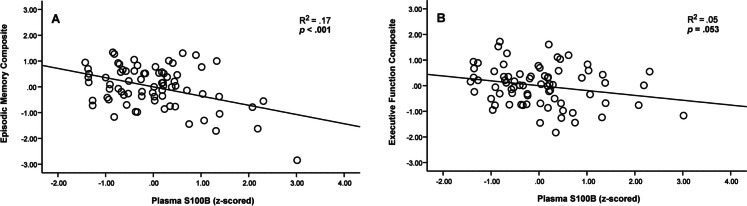
**Relationships between plasma S100b and cognition**. Regression plots for plasma S100B with memory (**A**) and executive function (**B**). Covariates in both models included age, sex, and education. Z-scored values are reported in the figures

A relationship was present between S100B and episodic memory performance (F(4,69) = 4.90, *p* = 0.002, S100B *β* = − 0.031, SE = 0.008, *p* < 0.001, *f* ^2^ = 0.205) after controlling for age, sex, and education. Higher levels of S100B were associated with poorer memory performance. Results for memory remained significant following Holm-Bonferroni correction. A similar negative relationship between S100B and executive function was found but only approached significance and did not survive multiple comparison correction (F(4,69) = 4.98, *p* = 0.001, S100B *β* = − 0.016, SE = 0.008, *p* = 0.053, *f* ^2^ = 0.057). Results are displayed in Fig. 3 and Table 2.

**Table 2:** The effect of S100B on cognitive and neuroimaging measures

Dependent variables	*n*	*F*-statistic	*β*	SE	95% CI [LL, UL]	*p*-value	*f* ^2^
**Cognitive domains**^a^							
Memory	**74**	4.90**	** − .031**	**.008**	**[− .047, − .014]**	** < .001**	**.205**
Executive function	74	4.98**	** − **.016	.008	[**− **.033, .000]	.053	.057
**k**_**w**_ **regions of interest**^b^							
Frontal lobe	62	1.81	.001	.003	[**− **.005, .007]	.739	.002
Parietal lobe	62	1.27	** − **.005	.003	[**− **.011, .002]	.173	.034
Hippocampus	56	0.98	.001	.009	[**− **.017, .018]	.941	.001
Left precuneus	62	2.54*	** − **.005	.008	[**− **.021, .010]	.503	.007
Right precuneus	62	2.22	.000	.009	[**− **.018, .017]	.953	.000
**White matter hyperintensities**^c^							
Total volume	71	8.30**	.002	.005	[**− **.008, .012]	.711	.003
Deep volume	71	6.73**	.003	.008	[**− **.013, .019]	.736	.001
Periventricular volume	71	7.97**	.003	.005	[**− **.007, .013]	.543	.006

S100B was measured in pg/mL. Estimates listed reflect S100B in the model

*SE* standard error, *CI* confidence interval, *LL* lower limit, *UL* upper limit

^*^p < .05; **p < .01

^a^Covariates: age, gender, educations

^b^Covariates: age, gender, region of interest size

^c^Covariates: age, gender, estimated intracranial volume

### Multiple Linear Regression: S100B and k_w_

Plasma S100B was not associated with k_w_ in the frontal lobe, parietal lobe, hippocampus, left precuneus, or right precuneus after controlling for age, sex, and ROI size (*p*s > 0.05, see Table 2).

### Multiple linear regression: S100B and WMHs

Plasma S100B was not associated with total, deep, or periventricular WMH volume controlling for age, sex, and estimated intracranial volume (*p*s > 0.05, see Table 2).

### Moderation analysis: S100B × k_w_

**Fig. 4:**
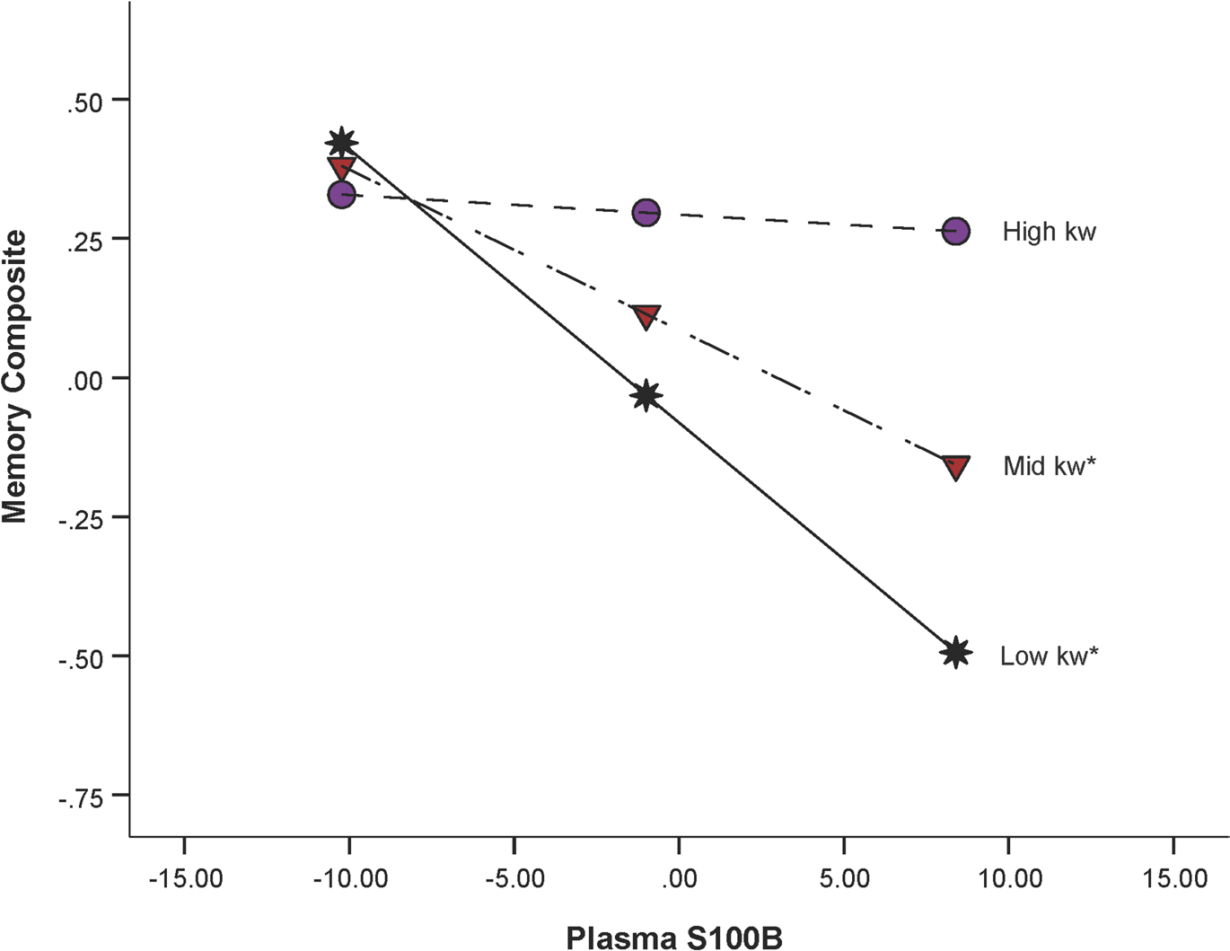
**Plasma S100b × parietal k**_**w**_ **moderation effect for episodic memory performance**. Plots for S100B and memory at high (purple circle), mid (red triangle), and low (black star) levels of the moderator (parietal lobe k_w_). Plots were created using the pick-a-point method, where low, mid, and high values represent 1 standard deviation below the mean, the mean, and 1 standard deviation above the mean of parietal lobe k_w_, respectively. The memory composite was z-scored. S100b was mean centered prior to analyses and is reported in pg/mL. *p < .05

Potential interactions between S100B and k_w_ on episodic memory performance and executive function performance were explored. A significant S100B × parietal lobe k_w_ interaction was found for episodic memory performance (F(7,54) = 3.55, *p* = 0.003; interaction *β* = 0.095, SE = 0.042, *p* = 0.028), after controlling for age, sex, education, and ROI size (see Fig. 4 and Table 3). When probing the interaction using the pick-a-point method, we found that having higher S100B was associated with poor memory performance in individuals with low parietal lobe k_w_ values (*β* = − 0.049, SE = 0.013, *p* < 0.001) and in those with mid parietal lobe k_w_ values (*β* = − 0.029, SE = 0.010, *p* = 0.005). In contrast, S100B did not impact memory performance in those with higher parietal k_w_ levels (*β* = − 0.004, SE = 0.016, *p* = 0.820). No interactions between S100B and k_w_ in any other ROIs were observed on memory performance [frontal lobe (interaction *β* = 0.005, SE = 0.042, *p* = 0.907), left precuneus (interaction *β* = − 0.005, SE = 0.015, *p* = 0.741), right precuneus (interaction *β* = 0.009, SE = 0.016, *p* = 0.593) or hippocampus (interaction *β* = 0.004, SE = 0.017, *p* = 0.826)]. Additional information on these null findings are reported in Supplementary Table 1. Additionally, moderation was not present between any of the five k_w_ ROIs (frontal lobe, parietal lobe, left and right precuneus, hippocampus) and executive function (*p*s > 0.05).

**Table 3:** Moderation analyses results for memory performance

Moderator: parietal k_w_ (*n* = 62)				
	***β***	**SE**	**95% CI [LL, UL]**	***p*-value**
Age	.004	.018	[**− **.032, .040]	.827
Gender	.375	.204	[**− **.034, .784]	.072
Education	.009	.036	[**− **.064, .081]	.811
ROI size	.000	.000	[**− **.001, .001]	.725
S100B	** − .028**	**.010**	**[− .048, − .008]**	**.006**
Parietal k_w_	.780	.401	[**− **.025, 1.58]	.057
S100B × parietal k_w_	**.095**	**.042**	**[.011, .179]**	**.028**
Moderator: deep WMHs (*n* = 71)				
	***β***	**SE**	**95% CI [LL, UL]**	***p*-value**
Age	.012	.018	[**− **.023, .047]	.499
Gender	**.619**	**.222**	**[.175, 1.06]**	**.007**
Education	.028	.034	[-.040, .095]	.423
eICV	.000	.000	[.000,.000]	.274
S100B	** − .030**	**.008**	**[− .046, − .015]**	** < .001**
Deep WMHs	** − .258**	**.122**	**[− .502, − .015]**	**.038**
S100B × deep WMHs	** − .025**	**.009**	**[− .043, − .007]**	**.007**

*SE* standard error, *LL* lower limit, *UL* upper limit, *ROI* region of interest, *eICV* estimated intracranial volume, *WMH* white matter hyperintensity

### Moderation analyses: S100B × WMHs

**Fig. 5:**
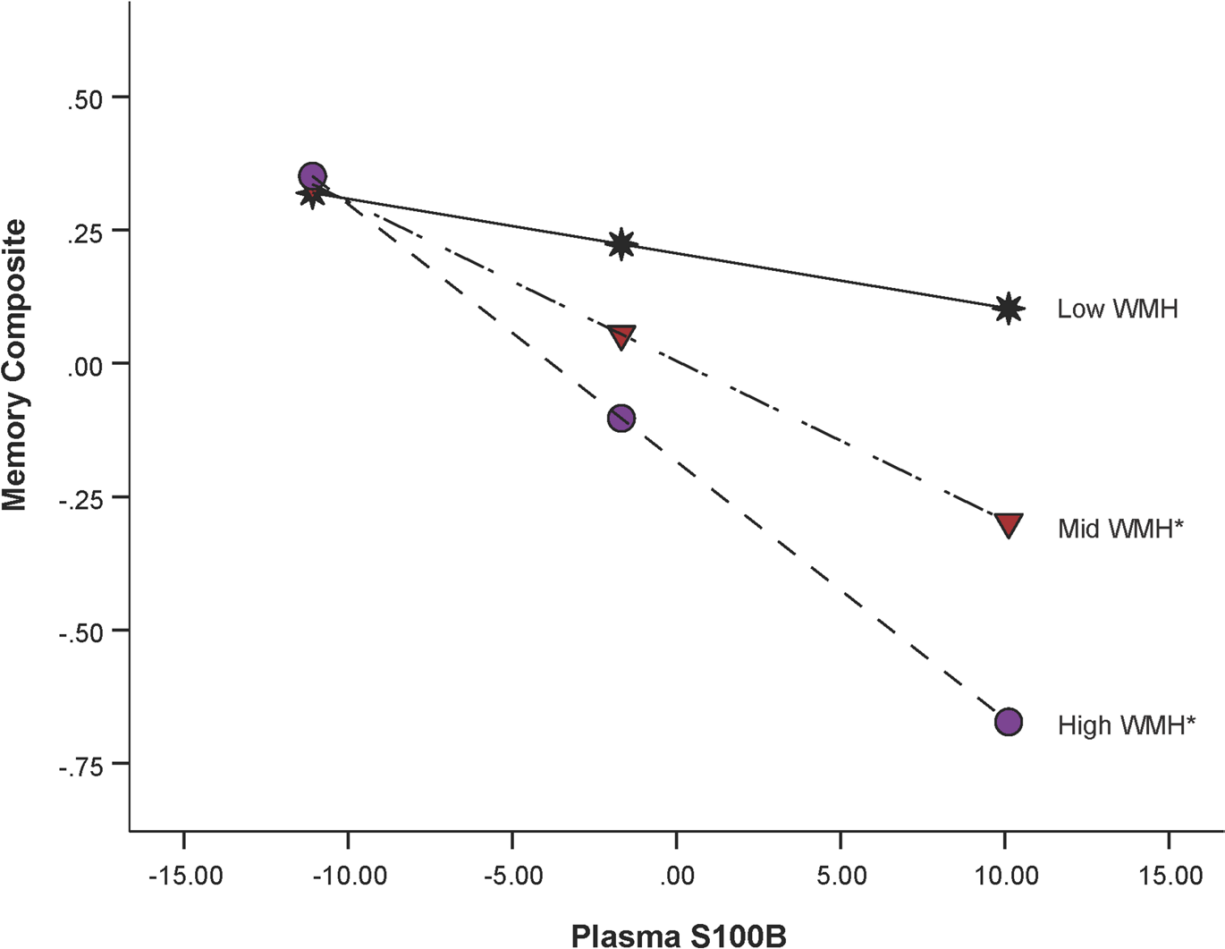
**Plasma S100b × deep white matter hyperintensity moderation effect for episodic memory performance**. Plots for plasma S100B and memory at low (black star), mid (red triangle), and high (purple circle) levels of the moderator (deep white matter hyperintensities). Plots were created using the pick-a-point method, where low, mid, and high values represent 1 standard deviation below the mean, the mean, and 1 standard deviation above the mean for deep white matter hyperintensities, respectively. The memory composite was z-scored. S100B was mean centered prior to analyses and is reported in pg/mL. *p < .05

Interactions between S100B and WMHs (total, deep, periventricular) were tested for episodic memory and executive function. A S100B × deep WMH volume interaction was found for memory performance (F(7,63) = 4.92, *p* < 0.001; interaction *β* = − 0.025, SE = 0.009, *p* = 0.007) when controlling for age, sex, education, and estimated intracranial volume (see Fig. 5 and Table 3). When probing the interaction, higher S100B levels were associated with poorer episodic memory performance for individuals in the mid (*β* = − 0.030, SE = 0.008, *p* < 0.001) to high (*β* = − 0.048, SE = 0.010, *p* < 0.001) WMH categories. For those within the low WMH category (*β* = − 0.010, SE = 0.010, *p* = 0.337), S100B did not further explain the association observed with episodic memory. No other interactions between WMHs and episodic memory were observed [total WMH (interaction *β* = − 0.024, SE = 0.015, *p* = 0.104), periventricular (interaction *β* = − 0.012, SE = 0.015, *p* = 0.139)]. Additional details for the null findings can be found in Supplementary Table 2. Finally, moderation was not observed for total, periventricular, or deep WMHs and S100B when examining executive function (*p*s > 0.05).

## Discussion

We investigated the relationships between plasma S100B levels, multiple MRI measures associated with cerebrovascular disease (CVD), and cognition in a community-based sample of older adults without dementia. Our results indicated that higher levels of plasma S100B were associated with poorer episodic memory performance. Further, this relationship was moderated by neuroimaging measures of BBB function (parietal BBB k_w_) and CVD (deep WMH volume). Specifically, the S100B-episodic memory associations were strongest in older adults with less efficient water exchange across the BBB (low to mid BBB k_w_) or greater WMH volume. Taken together, our findings suggest that high levels of plasma S100B are associated with lower memory performance in older adults, especially in those with greater CVD burden.

### Plasma S100B was negatively associated with cognitive performance in older adults

Our results were consistent with a previous report of poorer cognitive performance in older adults with higher levels of serum S100B levels [58]. Several other studies have also described negative associations between serum S100B levels and global cognitive measures in individuals with vascular risk factors [59, 60]. In contrast to the data presented, a single study demonstrated that higher serum S100B levels are associated with better performance on speed-based and executive function tasks [61]. It should be noted, however, that this latter study included a wider age range (range = 43–84 years old) than other work in this area and reported modest effects. While the difference in the direction of the relationships reported could be attributed to the multifunctional nature of S100B [(i.e., protective at low concentrations, harmful at high concentrations; see Donato et al. [62] and Michetti et al. [63] for review)], it could also be due to different levels of CVD burden across the participant samples studied.

### Relationships between plasma S100B and MRI measures of CVD

Our results that plasma S100B levels were not correlated with either of the neuroimaging metrics explored here (BBB k_w_ or WMH volume) are not surprising as the measures studied may reflect different components of VCID. Previous work suggests that S100B may be indicative of BBB leakage [14], astrocytic reactivity [64], and neuroinflammation [64, 65]. In comparison, BBB water exchange rate (BBB k_w_) is thought to be more related to glymphatic clearance functions than BBB leakage [21, 25, 26]. For instance, BBB k_w_ is only weakly correlated with BBB permeability (*K*_*trans*_) as estimated from dynamic contrast enhanced (DCE) MRI [21]. Similarly, while WMHs may partially reflect BBB damage [32], a shared feature with S100B levels, WMHs also reflect numerous other factors such as axonal degeneration and demyelination [28, 30, 66, 67]. In keeping with these differences, previous studies have found that S100B is not directly related to WMHs cross-sectionally [68] or with change in WMH burden over time [69].

### Synergistic effects of plasma S100B and MRI measures of CVD on cognition

A key finding of this study was the interaction between plasma S100B levels and MRI markers of CVD on episodic memory performance. Specifically, the negative effects of high S100B levels on memory performance were strongest in those with low to mid-range BBB k_w_ in the parietal lobe. Previous work by our group has shown that lower BBB k_w_ in cortical regions is generally associated with poorer cognitive performance [26, 27] and lower CSF amyloid beta (Aβ) 42 concentration levels (i.e., higher cerebral binding) in healthy older adults [26]. These previous findings are consistent with a view that low BBB k_w_ may in part reflect reduced glymphatic clearance of toxic proteins such as Aβ [26, 70].

Consequently, the interaction we observed suggests that elevated plasma S100B could be synergistically associated with lower BBB k_w_, further worsening glymphatic clearance functions. As noted, high levels of S100B may be representative of astrocytic damage and BBB leakage [14, 64], whereas low BBB water exchange rate (k_w_) may be indicative of reduced glymphatic clearance mechanisms, including disruption of aquaporin 4 (AQP4) channels that reside at the BBB-attached astrocytic end-feet [21, 71]. Additionally, S100B expression increases when AQP4 channels are inhibited [72]. This potentially synergistic, disruptive relationship could further reduce brain clearance mechanisms which, in turn, could negatively impact cognitive function [71, 73, 74].

The interaction we observed between plasma S100B and WMH volume on episodic memory performance suggests that S100B-associated astrocytic damage and/or BBB leakage could potentially further disrupt signaling in brain networks already partially disconnected by WMH-related (axonal/myelin) damage [75–77]. While WMH volume is associated with poorer cognitive function, effect sizes vary considerably across studies due to age, disease severity, and cognitive status [78–80]. The present results suggest that S100B levels, not measured in these prior studies, could be another contributor to the variability in the strength of relationship between WMH volume and cognition. At present, this and other potential relationships between CVD imaging biomarkers and S100B discussed above remain speculative. Further research will be required to address these potential relationships and to identify the combination of biofluid and neuroimaging markers that most accurately identify early-stage VCID, recognizing that different associations may be seen in different subpopulations with varying risk factors and underlying disease mechanisms.

### Region-specific findings for k_w_ moderation

Our region-specific finding for parietal k_w_ also warrants further comment. We did not find a moderation effect of k_w_ in the frontal lobe, precuneus, or hippocampus for the S100B-memory results. While episodic memory is often associated with the hippocampus [81, 82], the parietal lobe also contributes to memory processes. The parietal lobe is involved in episodic memory recall [83, 84], working memory [85], and integration of content corresponding to a given memory [86, 87]. It is therefore plausible that the memory measures used in our study, which were related to story recall and visuospatial recall, could be more susceptible to BBB k_w_ alterations in the parietal lobe than the hippocampus.

### Strengths and limitations

Strengths of this work include the multimodal approach to studying VCID. Specifically, our results suggest that the combined use of biofluid and neuroimaging markers may be more optimal than their separate usage in the early detection of VCID. Further, we examined novel biofluid and neuroimaging measures not under investigation by MarkVCID2, the validation phase of a multi-site US consortium devoted to developing biomarkers of VCID (https://markvcid.partners.org). Finally, our results add to the growing search for vascular (V) biomarkers to add to the canonical ATN framework for assessing age-related cognitive impairments as has been incorporated into the recently updated Alzheimer’s Association Criteria for AD [88].

Limitations of the study include the cross-sectional design, which limits causal inference of the associations seen. The sample was also primarily White, highly educated individuals, which limits the generalizability of the results. Additional studies among more diverse populations are needed to substantiate our findings. Further, we were unable to test three-way interactions between S100B, kw, and WMHs on cognition due to sample size constraints. It will also be important to examine longitudinal change in cognition as it relates to S100B levels and if the moderation effects we observe are also seen over time.

### Conclusion

In conclusion, our results support the combined use of non-invasive biofluid and neuroimaging in the study of VCID. While a direct negative relationship between S100B and episodic memory function was observed, the inclusion of neuroimaging measures revealed that high S100B was most strongly related to cognitive function in those with high CVD burden. Future work should identify the combination of markers most sensitive and specific to VCID for potential use in emerging clinical trials.

## Supplementary Information

Below is the link to the electronic supplementary material.

Supplementary file1 (DOCX 21 KB)
