# Supplementary material for: Synergistic effects of plasma S100B and MRI measures of cerebrovascular disease on cognition in older adults
Source: GeroScience. 2025 Feb 5;47(3):3131–46. doi: 10.1007/s11357-024-01498-1 (PMC12181517; doi:10.1007/s11357-024-01498-1)
Supplement: Supplementary file 1 — Supplementary file1 (DOCX 21 KB) [file 11357_2024_1498_MOESM1_ESM.docx]

Supplementary Table 1. Moderation k_w_ Regions of Interest

| **Moderator: Frontal k_w_ (n = 62)** | | | | | | |
| --- | --- | --- | --- | --- | --- | --- |
|  |  | **β** | **SE** | **95% CI**  **[LL, UL]** | **p-value** |  |
| Age |  | -.001 | .019 | [-.040, .037] | .945 |  |
| Gender |  | .332 | .220 | [-.110, .773] | .138 |  |
| Education |  | -.002 | .039 | [-.081, .077] | .964 |  |
| ROI size |  | .000 | .000 | [-.001, .001] | .744 |  |
| **S100B** |  | **-.032** | **.010** | **[-.053, -.012]** | **.003** |  |
| Frontal k_w_ |  | -.035 | .010 | [-.1.21, .951] | .944 |  |
| S100B x Frontal k_w_ |  | .005 | .042 | [-.080, .090] | .907 |  |
| **Moderator: Left Precuneus k_w_ (n = 62)** | | | | | | |
|  |  | **β** | **SE** | **95% CI**  **[LL, UL]** | **p-value** |  |
| Age |  | -.006 | .018 | [-.042, .029] | .719 |  |
| **Gender** |  | **.261** | **.199** | **[-.138, .659]** | **.196** |  |
| Education |  | .016 | .038 | [-.060, .091] | .683 |  |
| ROI size |  | -.016 | .000 | [.000,.000] | .274 |  |
| **S100B** |  | **-.038** | **.010** | **[-.058, -.017]** | **<.001** |  |
| Left Precuneus k_w_ |  | -.001 | .169 | [-.341, .338] | .994 |  |
| S100B x Left Precuneus k_w_ |  | -.005 | .015 | [-.026, .036] | .741 |  |
| **Moderator: Right Precuneus (n = 62)** | | | | | | |
|  |  | **β** | **SE** | **95% CI**  **[LL, UL]** | **p-value** |  |
| Age |  | .003 | .019 | [-.036, .042] | .879 |  |
| Gender |  | .365 | .200 | [-.036, .766] | .074 |  |
| Education |  | .002 | .040 | [-.078, .081] | .970 |  |
| ROI size |  | .006 | .009 | [-.011, .023] | .505 |  |
| **S100B** |  | **-.031** | **.010** | **[-.052, -.011]** | **.003** |  |
| Right Precuneus k_w_ |  | .095 | .159 | [-.224, .414] | .552 |  |
| S100B x Right Precuneus k_w_ |  | .009 | .016 | [-.023, .040] | .593 |  |
| **Moderator: Hippocampus (n= 56)** | | | | | | |
|  |  | **β** | **SE** | **95% CI**  **[LL, UL]** | **p-value** |  |
| Age |  | .008 | .019 | [-.031, .047] | .683 |  |
| Gender |  | .352 | .214 | [-.078, .782] | .106 |  |
| Education |  | .002 | .039 | [-.077, .081] | .963 |  |
| ROI size |  | .020 | .015 | [-.011, .050] | .202 |  |
| **S100B** |  | **-.031** | **.011** | **[-.052, -.009]** | **.006** |  |
| Hippocampus k_w_ |  | -.192 | .166 | [-.527, .142] | .253 |  |
| S100B x Hippocampus k_w_ |  | .004 | .017 | [-.030, .037] | .826 |  |

Note. SE = standard error. LL = lower limit. UL = upper limit. ROI = region of interest.

Supplementary Table 2. Moderation White Matter Hyperintensities

| **Moderator: Total WMH (n = 71)** | | | | | | |
| --- | --- | --- | --- | --- | --- | --- |
|  |  | **β** | **SE** | **95% CI**  **[LL, UL]** | **p-value** |  |
| Age |  | .022 | .018 | [-.014, .059] | .228 |  |
| **Gender** |  | **.683** | **.219** | **[.245, 1.12]** | **.003** |  |
| Education |  | .026 | .033 | [-.041, .093] | .438 |  |
| eICV |  | .000 | .000 | [.000, .000] | .095 |  |
| **S100B** |  | **-.029** | **.008** | **[-.045, -.013]** | **.001** |  |
| **Total WMH** |  | **-.591** | **.189** | **[-.968, -.214]** | **.003** |  |
| S100B x Total WMH |  | -.024 | .015 | [-.054, .005] | .104 |  |
| **Moderator: Periventricular WMH (n = 71)** | | | | | | |
|  |  | **β** | **SE** | **95% CI**  **[LL, UL]** | **p-value** |  |
| Age |  | .021 | .018 | [-.015, .058] | .242 |  |
| **Gender** |  | **.672** | **.219** | **[.234, 1.11]** | **.003** |  |
| Education |  | .029 | .033 | [-.038, .096] | .394 |  |
| eICV |  | .000 | .000 | [.000,.000] | .105 |  |
| **S100B** |  | **-.028** | **.008** | **[-.044, -.012]** | **.001** |  |
| **Periventricular WMH** |  | **-.613** | **.195** | **[-1.00, -.223]** | **.003** |  |
| S100B x Periventricular WMH |  | -.012 | .015 | [-.054, .008] | .139 |  |

Note. SE = standard error. LL = lower limit. UL = upper limit. eICV = estimated intracranial volume. WMH = white matter hyperintensity.
